# Cardiovascular Disease and Possible Ways in Which Lycopene Acts as an Efficient Cardio-Protectant against Different Cardiovascular Risk Factors

**DOI:** 10.3390/molecules27103235

**Published:** 2022-05-18

**Authors:** Ming-Ju Hsieh, Chih-Yang Huang, Rudolf Kiefer, Shin-Da Lee, Nancy Maurya, Bharath Kumar Velmurugan

**Affiliations:** 1 Oral Cancer Research Center, Changhua Christian Hospital, Changhua 50006, Taiwan; 170780@cch.org.tw; 2 Department of Post-Baccalaureate Medicine, College of Medicine, National Chung Hsing University, Taichung 40227, Taiwan; 3 School of Medicine, Chung Shan Medical University, Taichung 40201, Taiwan; 4 Department of Medical Laboratory Science and Biotechnology, Asia University, Taichung 41354, Taiwan; cyhuang@mail.cmu.edu.tw; 5 Cardiovascular and Mitochondrial Related Disease Research Center, Hualien Tzu Chi Hospital, Buddhist Tzu Chi Medical Foundation, Hualien 970473, Taiwan; 6 Graduate Institute of Biomedical Sciences, China Medical University, Taichung 40402, Taiwan; 7 Center of General Education, Buddhist Tzu Chi Medical Foundation, Tzu Chi University of Science and Technology, Hualien 970302, Taiwan; 8 Department of Medical Research, China Medical University Hospital, Taichung 40402, Taiwan; 9 Conducting Polymers in Composites and Applications Research Group, Faculty of Applied Sciences, Ton Duc Thang University, Ho Chi Minh City 758307, Vietnam; rudolf.kiefer@tdtu.edu.vn; 10 Department of Physical Therapy, Asia University, Taichung 41354, Taiwan; 11 Department of Physical Therapy, Graduate Institute of Rehabilitation Science, China Medical University, Taichung 406040, Taiwan; 12 School of Rehabilitation Medicine, Weifang Medical University, Weifang 261053, China; 13 Botany Department, Government Science College, Pandhurna, Chhindwara, M.P., Pandhurna 480334, India; nm_7582@rediffmail.com; 14 Faculty of Applied Sciences, Ton Duc Thang University, Ho Chi Minh City 758307, Vietnam

**Keywords:** lycopene, cardiovascular disease, pharmacokinetics, in vitro studies, in vivo studies

## Abstract

Foods rich in antioxidants such as lycopene have a major role in maintaining cardiac health. Lycopene, 80% of which can be obtained by consuming a common vegetable such as tomato, can prevent the disturbances that contribute to cardiovascular disease (CVD). The present work begins with a brief introduction to CVD and lycopene and its various properties such as bioavailability, pharmacokinetics, etc. In this review, the potential cardio-protective effects of lycopene that reduce the progression of CVD and thrombotic complications are detailed. Further, the protective effects of lycopene including in vitro, in vivo and clinical trials conducted on lycopene for CVD protective effects are explained. Finally, the controversial aspect of lycopene as a protective agent against CVD and toxicity are also mentioned.

## 1. Introduction

Cardiovascular diseases (CVD) affect a large percentage of people all over the world. Cardiovascular pathogenesis is a complex process, induced by certain ischemic factors with additional complementary factors such as high levels of blood pressure, endothelial dysfunction, lipid levels, etc. [1]. Among all the risk factors, diet remains the most crucial and modifiable factor that can be modulated in many ways for primary prevention of CVD. This is evident from the fact that CVD mortality rate is comparatively low in Mediterranean countries due to their healthy dietary habits with high fruit and vegetable amounts, particularly tomato [2].

Cardiovascular health is heavily influenced by an individual’s dietary habits and is quite valuable for planning nutritional strategies to deal with specific metabolic risk factors associated with cardiovascular health. Studies show that consumption of tomatoes and lycopene supplements has critical health benefits and significantly reduces LDLcholesterol along with lowering systolic blood pressure. This indicates the positive association between tomato and low CVD risk [3]. Since tomatoes are a rich source of lycopene (Table 1), including them regularly in fairly good amounts in the diet may help to combat cardiovascular disease risk factors.

## 2. Dietary Sources of Lycopene

Fruits and vegetables remain the major lycopene source with the majority share being provided by tomatoes and processed tomato products such as ketchup [4]. Bitter melon, rosehip, red carrot and autumn olives are the best new sources of lycopene [5], of which autumn olives have 5 to 20 times more lycopene content than tomato [6]. Several factors influence the lycopene content of fruits and vegetables, such as environmental conditions (temperature, irrigation, light, climate, location of plantation), fruit variety, degree of ripeness, processing and storage conditions [7]. The lycopene level increases in ripening fruits, as chlorophyll level tends to decrease.Factors such as light and oxygen were found to be the most influencing factors for the processing and storage of lycopene products. It has been found that supplementation with lights such as red and blue promotes lycopene synthesis in tomatoes [8]. Lycopene level in tomato paste was found to be the most stable compound (with 20% total loss) as compared to other compounds such as ascorbic acid, quercetin, and kaempferol, even after repeated sterilization and evaporation [9]. The presence of phenolic compounds, ascorbic acid, etc. may be responsible for the stability of lycopene and its presence in tomato fruit, including matrices such as isomerization, autoxidation, etc. In addition, they provide more stability as compared to the pure forms of lycopene [10].

Studies on the storage stability for tomato juice show that post-processing stability is influenced by factors such as storage, time, variety, processing method [11], etc. In addition, owing to its hydrophobic nature, lycopene is more available in processed forms as compared to raw tomatoes [12]. Sources of lycopene food and its content level as per the USDA 2019 are tabulated in Table 1.

## 3. Lycopene Biochemistry

**Figure 1:**
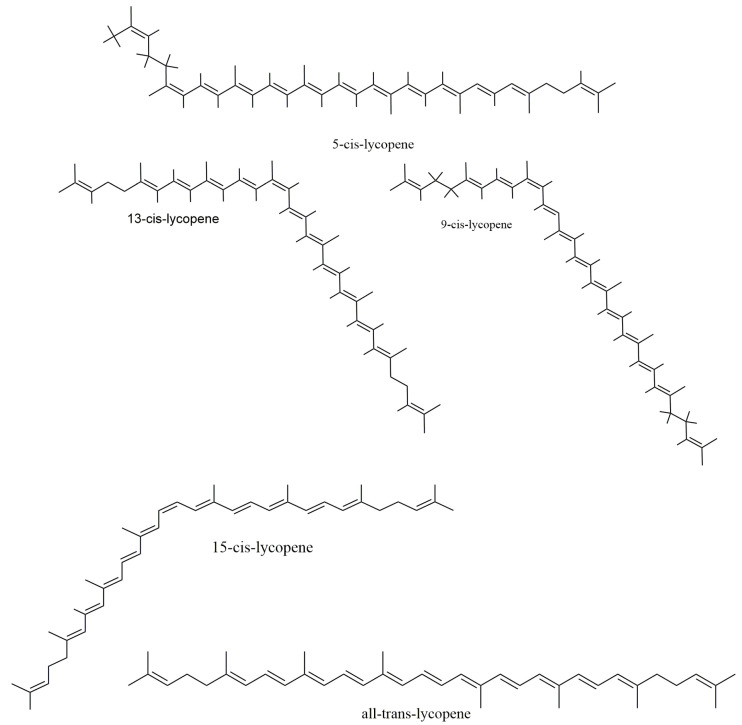
Chemical structures of lycopene.

Carotenoids, found in a fairly good amount in tomatoes, are colorful lipid molecules found extensively in the leaves, fruit, roots and tubers. These are divided into three groups, namely xanthophylls, carotenes and lycopene. More than 700 carotenoids are found in nature [13]. Lycopene is a tetra-terpene from the carotene sub-group composed of carbon and hydrogen (C_40_H_56_) with 11 linearly conjugated double bonds. The structure of lycopene (Figure 1) is considered as the prototype of most carotenoids, as most structures can be related to that of lycopene’s through structural modifications.

Natural occurrence of lycopene shows that it exists mostly as a *trans*-isomer [14], while in tissues and plasma, it appears as a *cis*-isomer [15]. A total of 2048 isomers (that is, 2^11^) is possible if each of the 11 carbon–carbon double bonds undergoes isomerization, producing a cluster of mono- and poly-*Z*-isomers. However, a culmination of this cluster formation is made by steric hindrance, as some ethylenic groups can only isomerize from *E*- to *Z* forms. In the presence of light, heat exposure and certain biochemical reactions, all (*E)*-form isoforms are converted to (*Z*)-isoforms. Upon isolation, all conjugated (*E*)isoforms areunstable and undergo oxidation upon exposure to light, high pH and temperature. However, this demerit of instability does not exist for lycopene inside the tomato matrix due to probable protection by the cell membrane. Thus, apart from being a natural dietary source of lycopene, it also stays stable in the tomatoes in their natural form.

## 4. Dietary Metabolism and Retention of Lycopene Bioavailability

Post release of lycopene into the duodenum after consumption of lycopene-rich foodsforms complexes by combining with bile and fatty acids, which are known as lycopene micelles. These micelles have hydrophilic shells, with lycopene containing a hydrophobic core, which is then absorbed and packed into the small intestine, forming chylomicrons through a passively controlled diffusion method. They are then delivered into the bloodstream and lymphatic system and then finally delivered into the liver and other organs. Lycopene is among those carotenoids whose concentrations are found to be high in the prostate, testes and adrenal glands [16].

Processing of tomato promotes the release of lycopene from its binding site, and the process of adding oils makes the lycopene more abundant for micelles formation followed by intestinal absorption [17]. Thus, any factor such as drug, pathogenic condition, intake of dietary compounds, which may cause mal-absorption of lipid, maywell disturb this process of micelles formation, thereby reducing the bioavailability of lycopene.

Dietary composition greatly influences the bioavailability of lycopene for its lipid-soluble nature, and thus, consuming it along with fat enhances it bioavailability. As for instance, full-fatdressed salads lead to enhanced levels of blood carotenoid level as compared to salads with less-fat dressing. This study shows that consuming salad without fat dressing has no major lycopene uptake [18]. Another study showed that tomato salsa consumption along with avocado leads to a 4.4-fold increase in absorption of lycopene as compared to ones without using avocado as the lipid source [19].

It has been absolutely difficult to establish the oral lycopene fate in humans as observed in plasma. An AMS (accelerator mass spectrometry) study showed that after ingestion, all *trans*-isomers were converted into *cis*-isomers, and post absorption of lycopene or lycopene metabolites were transferred to the skin. In addition, lycopene in part is metabolized through *β*-oxidation, converting into polar metabolites and carbon dioxide, which are excreted in the urine. In plasma, both ^14^C-lycopene and unlabeled lycopene follows first-order kinetics [20]. It has been proposed that absorption of *cis*-isomers of lycopene is preferred over *trans*-isomers, and isomerization of *trans*-isomers occurs in the intestinal lumen [21], liver or enterocytes [22]. This study also showed that extensive isomerization of all-*trans*-isomers occursin vivo, which provides the evidence that humans can isomerize all-*trans*-isomers, particularly 13- and 15-isomers.

## 5. Lycopene Pharmacokinetics

### 5.1. Absorption

Being a fat-soluble compound, lycopene and its absorption pattern are similar to dietary fat, which is separated from the food matrix inside the stomach and duodenum and is dissolved in the liquid phase [23]. Lipid droplets are formed prior to absorption, which results from the interaction of pancreatic lipases and bile salts. These droplets look similar to multi-lamellar lipid vesicles upon entering the duodenum, which are absorbed in the small intestines through passive diffusion [24]; however, studies have shown that absorption is also helped by epithelial transporters [25]. In this section, we reviewed Lycopene Pharmacokinetics using Caco-2 cells, because this is derived from intestinal tissue. An in vitro study conducted on Caco-2 cells showed that lycopene absorption is low as compared to other carotenoids [26]. Many factors influence the absorption rate of lycopene, viz. increased indigestible fraction decreases lycopene release from the food matrix into the digestive tract [27], reduced lycopene uptake exists in diets high in fiber, and plasma lycopene decreases by around 40% when dietary fibers are included in a lycopene supplemented diet [28]. Bio-accessibility of lycopene is greater in the large intestines (57%), as compared to the small intestines (40%), but the absorption amount is negligible in the large intestines and the *trans*-isomer is taken up relatively in higher amounts as compared to the *cis*-forms, as observed in Caco-2 cells [29].

In human plasma, 60% of *cis*-isomers are isomerized, and even *trans*-lycopene is rich (>90%) in a lycopene diet [30]. The acidic condition within the gastric milieu enhances this process, which further improves lycopene absorption in the small intestines [31].

### 5.2. Transportation

Lycopene is transported to triacylglycerol-rich chylomicrons post uptake by intestinal mucosa, which is secreted into the lymphatic system and finally reaches the liver [32]. Lycopene is deposited in the membrane within the lipophilic compartment or lipoprotein, which is transported by lipoproteins of plasma, and the chemical structure of lycopene determines the distribution. Owing to its hydrophobic nature, lycopene is present in the core region of a lipoprotein, i.e., the lipophilic part for which low-density lipoproteins mainly transport lycopene. As compared to *trans*-isomers, *cis*-isomers have a higher probability of incorporating into lipoproteins due to their shorter chain lengths [33].

### 5.3. Distribution

Lycopene is found in higher concentrations (ten times higher) in the liver, adrenal glands, and reproductive tissue [34] and includes, in order of concentration, the testes, adrenal glands, prostate, breast, pancreas, skin, colon, ovary, lung, stomach, kidney, fat tissue and cervix. Plasma lycopene may vary from people of different countries such as in Italy (Ragusa/Naples) [35], which is highest in males (1.29 ± 0.46 µmol/L) and females (1.32 ± 0.46 µmol/L) and lowest in United Kingdom females [36]. Eating behavior among individuals also affects lycopene level, and this variation has been observed in unmarried, married and divorced individuals [37].

## 6. Cardio-Vascular Disease Risk Factors

There are many risk factors that underlie the induction and progress of CVD in an individual. Crucial CVD parameters for this complex pathophysiology are discussed below:

### 6.1. Oxidation of LDL (Low Density Lipoprotein)

A central role is played by oxidative stress in CVD either in pathophysiology after extended ischemia or in primitive lesions [38]. Evidence in animal models shows that oxidative modification of LDL has great involvement in the pathogenesis of atherosclerosis [39], which is a chronic inflammatory condition playing as a major cause of CVD (Frostegård, 2013) and is a well-known risk marker for CVD (Itabe, 2009). According to Itabe (2009), oxidized low-density lipoproteins (OxLDL) have been found to exhibit pro-atherogenic properties in vitro.In vivo studies have shown occurrence of OxLDL in atherosclerotic lesions and plasma specimens of atherosclerosis patients (Itabe, 2009). Further, clinical studies have also revealed the importance of plasma OxLDL measurement, but metabolism, behavior and modified structure of OxLDL are not completely understood (Itabe, 2009). Evidence from recent investigations support multiple LDL modification theory and suggests that LDL particles undergo many modifications that alter their density, size, chemical properties in blood flow and vascular wall, while oxidation is the last stage in the whole cascade, which results in atherosclerogenic properties (Poznyak et al., 2021). Oxidized LDL can activate macrophages and other cells triggering inflammation; however, some recent investigations have also discovered that OxLDL may have both pro- and anti-inflammatory properties (Poznyak et al., 2021).

### 6.2. Involvement of Metal Ions in LDL Oxidation

Metal ions remain to be one of the most critical players for oxidation of LDLas lipid hydroperoxides (Girotti, 1998). Hydroperoxides are the first products of lipid oxidation, which undergo cleavage (carbon-carbon) aided by alkoxyl radicals forming unesterified short-chain aldehydes in the presence of transition metals [40]. A protein, which is important for iron and hemoglobin turnover, called haptoglobin and haptoglobin 2-2 genotype, is related to a 2–5-fold increase in CVD due to increased oxidative stress [41]. There have been studies explaining the mechanism involved in copper- and iron-dependent oxidation modification of LDL, the causal step in atherosclerosis (Lynch and Frei, 1993). However, the mechanism of LDL oxidation is still unclear (Lynch and Frei, 1993; Itabe, 2009; Levitan et al., 2010).

### 6.3. Role of Enzymes in LDL Oxidation

Enzymes such as myeloperoxidase and lipoxygenases are linked toward oxidation of LDL. The former is the only enzyme present in humans capable of producing HOCl (hypochlorous acid), which is a cytotoxin from H_2_O_2_ (hydrogen peroxide) and chloride. 3-chlorotyrosine is an oxidation marker found in elevated levels in human LDL isolates [42]. Lipid peroxides are transferred to LDL, which are produced by intracellular lipoxygenase and released from cells [43]. Modifications of LDL are also carried out by other enzymes such as oxidase, lipase, etc. (Poznyak et al., 2021), while there are non-enzyme-mediated modifications as well, such as, interaction with free radicals, proteoglycans, glycosylation, etc. (Poznyak et al., 2021).

### 6.4. High Blood Sugar and Blood Pressure

Patients with diabetes mellitus and high blood pressure (BP) are at a high risk of CVD. A 70% increase in BP occurs in such people [44]. The more intensively treated patients had low incidence of stroke as compared to the lesser treated ones, and trials based on outcomes showed that a proportional relation between prevention of myocardial infarction (MI) and BP reduction exists. However, the magnitude of this reduction and risk of MI is not more significant, and the risk of MI and degree of BP reduction did not show any additional benefits [45].

## 7. In Vitro Studies-Based Evidence of Lycopene’s Protective Effect against CVD Factors

Many studies have been conducted for in vitroinvestigation of lycopene derived from foods and supplements in search of their risk-reducing effects on CVD. These studies were mostly dose dependent on different cell lines. A previous study showed that at 2–12 µmol/L lycopene can inhibit platelet aggregation via activation of cyclic GMP for nitrate formation and suppressing phospholipase C [46]. A significant decrease in LDL oxidation (copper catalyzed) at 0–200 µmol/L by lycopene on human plasma cells was observed, along with suppression of lipid peroxides and TBARS (thiobarbituric acid-reactive substances) [47]. Another study on HUVECs (human umbilical vein endothelial cells) and THP-1 monocytes indicated that lycopene can inhibit NF-κB activation induced by TNF-α together with ICAM-1 (Intracellular Cell Adhesion Molecules 1) and VCAM-1 (Vascular Cell Adhesion Molecules 1) expression and monocyte endothelial association. In addition, no significant expression of COX-2 (cyclooxygenase-2) and PECAM-1 (Platelet Endothelial Cell Adhesion Molecules 1) was reported in this study [48].

Lycopene also protects against H_2_O_2_ attacks and reduces apoptosis and p53 expression on Vascular endothelial cells at around 0.2–20 µmol/L [49]. Studies conducted by Palozza et al., 2010, [50] on macrophages (human THP-1) showed that at 0.5–2 µmol/L, lycopene significantly reduced ROS production and 7-KC induced apoptosis by subsequently suppressing caspase-3 activation. Another study by the same group showed that lycopene reduces the total cholesterol content through the expression of HMG-CoA reductase (3-hydroxy-3-methylglutaryl-coenzyme A) [51]. Further works in this same direction by Di Timo et al., 2012, [52] and Sung et al., 2015, [53] showed that lycopene is capable of reducing TNF-α inflammation and inhibiting ET-1 (Endothelin-1) expression together with HO-1 (Heme oxygenase-1) induction. These studies thus suggest that lycopene has an inevitable role toward CVD development, which will be further described from the in vivo studies described below.

## 8. In Vivo Studies-Based Evidence of Lycopene’s Protective Effect against CVD Factors

Most of the studies conducted in vivo considered lycopene in the form of supplements such as raw tomato, ketchup, puree, soup and lycopene capsules. The minimum time of administration in these studies was as low as 6 h, and the longest period went for 12 weeks. Range of dosage ran from 4 mg/day (8 weeks) to 80 mg/kg (1 week time). A decrease in lipo-oxidation of serum lipid and LDL oxidation was observed for 10 males and 9 females aged between 25–40 years for 1 week with a dosage of 39.2 mg/day (spaghetti sauce), 50.4 mg/day (tomato juice) and 75 mg/day (lycopene capsules) [54]. Similar results were also found by another study with tomato juice (40 mg/day) for 2 weeks on 23 male subjects aged 27–40 years [55].

However, no reduction or delay in LDL oxidation was observed for 47 subjects (25 females, 22 males) below 65 years for lycopene capsules with 13.3 mg/day for 12 weeks [56]. Similar results were observed for 175 male subjects with 15 mg/day for 12 weeks [57]. However, significant reduction in LDL oxidation was observed with no plasma antioxidant capacity. In a study with tomato products such as paste, raw and sauce with 12 females aged between 22–38 years with a dosage of 8 mg/day for 3 weeks found DNA damage reduction by 24% [58]. Lycopene treatment on SD (Sprague–Dawley) male rats (5–10 mg/kg body weight/day for 10 days) with hypertriglyceridemia and oxidativestress (LPS-stimulated) was found to have downregulation of PCSK-9 (proprotein convertase subtilisin/kexin type-9) expression through HNF-1α (hepatocyte nuclear factor) where the LDLreceptor was increased due to elevated regulation of sterol regulatory element-binding protein-2 [59]. Mice treated with lycopene (10 mg/kg) exhibited inhibition of the NF-κB pathway, which decreases apoptosis and inflammation of cardiomyocytes post-MI (Myocardial infarction) [60]. In a rat model, lycopene induced an elevation in MMP-9 (Matrix metalloproteinase), which is related to MI and expression of type I collagen. It also inhibited the activation of p38 with decreased collagen volume in the peri-infarcted zone [61]. One study with similar treatment showed that angiotensin-II-induced cardiovascular remodeling is prevented by inhibiting cardiac fibrosis, hypertension, hypertrophy, and vascular damages [62]. All these and many more in vivo studies have indicated lycopene’s effects on various metabolic pathways.

## 9. Clinical Trials-Based Evidence of Lycopene’s Protective Effect against CVD Factors

Mixed results were obtained from human interventional studies against cardio-protective effects of lycopene. A total of 54 studies were conducted between 1998–2010 using tomato-based products and lycopene supplements for assessing their effects on CVD risk factors of which 35 studies showed beneficial effects. Conventional markers of CVD such as CRP (C–reactive protein), blood pressure and serum concentration levels were included in 13 studies. Benefits of increased lycopene intake were observed in those studies, which included such non-established markers, viz. DNA damage, lipid peroxidation, LDL oxidation, inflammatory markers other than CRP, platelet activation, etc. Statistical observations were lacking in most studies due to the small number of volunteers (below 100). Further, a majority of trials (43 of 49) consisted of 30 days and were poorly controlled with varying lycopene sources and doses (tomato juice, puree, soup, supplements with 5–80 mg doses) [63].

Another study showed that increased lycopene consumption from tomato products is more effective than lycopene supplements for enhancing serum proteins and lipids and inflammatory markers such as CRP, while supplements are more effective in lowering blood pressure [64]. The presence of other components in tomatoes such as vitamin A, potassium, ascorbic acid and bioactive phytochemicals may contribute toward its effectiveness [65]. Daily consumption of 29.4 mg lycopene for 30 days reduced serum CRP in women with similar compliance within a pilot study of 40 heart failure patients (23 men, 17 women) [66]. A randomized, double blind, placebo-controlled intervention trial consisting of healthy volunteers and statin-treated CVD patient with a lycopene supplementation of 7 mg daily for 2 months showed a 53% improvement in arterial vasodilation (endothelial-dependent) for patients under secondary treatment (prevention), but no effect was observed in healthy volunteers [67].

Further, lycopene supplementation of 20 mg/d for 12 months increased the efficacy of lutein supplementation of 20 mg/d in decreasing the thickness (intima-media) of the carotid artery in 144 patients with sub-clinical atherosclerosis (0.073 mm decrease in thickness with both lycopene and lutein vs. 0.035 mm decrease with lutein alone) [68]. To estimate the effects of lycopene in tomatoes or supplements for modulation of CVD risks, there was a study in the UK with 225 volunteers comprising 94 men and 131 women between ages 40–65 years. They were divided into three groups based on dietary interventions: first a controlled diet (low consumption of tomato-based food), second a high tomato-based diet (30–50 mg lycopene/day) and a controlled diet supplemented with lycopene capsules (10 mg/day) for 12 weeks. Results obtained showed that none of the systematic CVD markers (insulin resistance and sensitivity markers, lipid concentrations, inflammatory markers) changed. Arterial stiffness and blood pressure were also unaffected [69]. Another study showed that increased lycopene intake by dietary means or byusing supplements for 12 weeks reduced HDL_3_ and serum amyloid A content [70].

## 10. Therapeutic Potential of Lycopene against Cardiac Risk Factors

Lycopene belongs to a broader family of lipid soluble antioxidants that is carotenoids, which are available in vegetables and fruits, particularly tomatoes. They possess the ability to finetune key events critical for CVD such as apoptosis, inflammation and cellular communications [71]. High oxidation potential of lycopene leads to a decrease in LDL (low density lipoprotein) and subsequently increases endothelial functioning, which further decreases the risk of CVD. A recent meta-analysis showed that lycopene intake is associated with a 17% reduction in risk of CVD [72].

Many studies have shown that lycopene has many cardio-protective effects against oxidative stress induction by ROS production [73], inhibition of stress from the endoplasmic reticulum initiated from myocardial reperfusion/ischemia injury [74], prevention of oxidative damage due to LDL [75], promotion of ventricular remodeling by preventing apoptosis [60] and improvement in endothelial functions [67]. Potential antioxidant properties of lycopene reduced prophylaxis progression and thrombotic progression and complications in atherosclerosis, which is carried out by the following six mechanisms: inhibition of endothelial injuries, prevention of cholesterol by prohibiting 3-hydroxy-3-methylglutaryl coenzyme A reductase, which acts as rate limiting enzyme for cholesterol synthesis, efflux promotion of cholesterol, decrease in pro-inflammatory activity of T lymphocytes and macrophages, and inhibition of foam cell production and smooth cell formation by influencing molecular pathways that control cell division and apoptosis [76].

It has been found recently that lycopene supplementation provides protective effects toward CVD risk; however, a consensus of all the results obtained from different studies could not be made. A general conclusion from these studies showed that consuming foods containing lycopene is more effective than lycopene supplements [77]. The probable cause for this may be the additional compounds such as bioactive molecules and electrolytes, which potentially elevate the bioavailability of lycopene and related effects [65]. Another study showed an inverse association with lycopene consumption and ischemic heart disease incidence along with coronary insufficiency [78]. The authors from a randomized trial of 225 people found a positive result of lycopene consumption from tomato-based products on inflammatory, lipid and vascular markers, and lycopene induced an improvement in HDL function [69].

Lycopene supplementation from diets (once per day for 1 week) through tomato juice (50.4 mg lycopene), tomato oleoresin (75.0 mg lycopene) and spaghetti sauce (34 mg lycopene) significantly increased serum lycopene. Lipid peroxidation in serum and LDL oxidation decreased post consumption of a lycopene-rich diet with no differences in levels of serum cholesterol [54]. However, in women, it was found that high levels of plasma lycopene was related to low levels of CVD [79]. The development of atherosclerosis in smokers is prevented by circulating plasma lycopene [80]. Formation of atherosclerotic plaques in the aorta are also controlled by plasma lycopene and improved the lipid profiles of rabbits with high-fat diet as compared to other controlled groups [81]. In hypertension patients, blood pressure was reduced by antioxidant-rich tomato juice extract (250 mg/day, for eight weeks) in a short-term treatment [82]. Thus, dietary lycopene may have preventive benefits on CVD, which may increase with increased intake of a lycopene-rich diet such as tomato sauce, etc. [83].

### 10.1. Lycopene on Oxidative Stress

Lycopene can show interactions with a huge range of ROS (reactive oxygen species), as lycopene has the highest _1_O^2^ quenching rate, wherein it can quench _1_O^2^ to an unreactive triplet ground state (_1_O^3^) giving out energy. Extreme hydrophobicity within the lipid bilayers of cell membranes (logP~14.5 at 25 °C) helps this quenching process by lycopene as it holds a place in the hydrophobic core of the cell membrane [84]. In addition, Lycopene’s antioxidant properties are well established; however, the underlying exact mechanism of its action is still unknown. There is some evidence that shows that lycopene protects against CVD by inhibiting TNF-α-induced NF-κB, expression of ICAM-1 and monocyte endothelial interaction (Hung et al., 2008), but human studies on the protective effects of lycopene toward CVD have given mixed results (35 of 54 intervention studies conducted between 1998–2010 foundgood effects on CVD risk factors) [85].

The positive effects of lycopene in controlling oxidative stress have been provenin vitro, exvivo and in vivo where oxidation of lipid, protein and DNA are related to oxidative stress. It has been reported that lycopene-rich diet and supplements provide protection against DNA damage in normal and cancerous cells [86]. An inverse association of plasma lycopene levels with oxidative stress is revealed [87]. In lymphocytes, it was found that lycopene-rich foods, supplements and juices showed protective effects against oxidative stress [88]. Lycopene consumption in the form of oleoresin capsules and ketchup in humans showed a decrease in lipid and protein oxidation [89]. Hormonal replacement therapy in most menopausal women can be replaced by using lycopene supplement (4 mg/day for six months) for preventing atherosclerosis and oxidative stress [90].

### 10.2. Anti-Diabetic Effects of Lycopene

Disturbed glucose metabolism and type2 diabetes is inversely associated with lycopene intake. A decrease in plasma glucose and concentration of fasting glucose were observed with an increase in serum lycopene [91]. Few evidence has found the relationbetween lycopene and diabetes-induced oxidative stress by measuring several biomarkers and lipid peroxidation products, including enzymatic endogenous antioxidants glutathione peroxidase (GPx), superoxide dismutase (SOD), and malondialdehyde (MDA) levels in the plasma or tissue samples [92,93]. Inold diabetic patients, plasma lycopene level decreases as compared to control individuals, and inverse relations were found between lycopene and age [94]. Dietary lycopene modulates IGF (insulin-like growth factor), and small amounts of lycopene and carotenoids also affect IGF-1 [95]. Few studies have found that lycopene consumption does not reduce diabetes mellitus type 2 risk [96]. Bose and Agrawal [97] found no significant changes in FBG and HbA1c levels for T2DM patients following a 30-day supplementation of ripe cooked tomatoes (200 g tomatoes/day). She et al. did not find a significant association between HbA1c and lycopene level in a sample of 40 T2DM participants [98]. Similarly, Upritchard et al. [99] supplemented 500 mL of tomato juice along with Vitamin E and C for 4 weeks for T2DM patients and observed that lycopene supplementation did not affect plasma glucose concentration.

### 10.3. Lycopene’s Effect on Flow-Mediated Dilation (FMD)

All blood vessels are internally lined by endothelial cells, and their disruption is termed as endothelial dysfunction that is associated with a pathophysiological role in CVD and atherosclerosis (Vita andKeaney, 2002). Several studies have shown that FMD is inversely proportional to CVD (Katz et al., 2005). The change in the diameter of the brachial artery with blood flow measures FMD, and it has been shown that an increase in 1% FMD leads to a decrease of 13% CVD (Inaba, Chen, & Bergmann, 2010). Considering the effect of lycopene supplementation on FMD, some human volunteer-based studies have reported that tomato paste increases FMD by ~3.3%, indicating that its daily consumption produces a beneficial mid-term effect on endothelial function (Xaplentaris et al., 2012); however, previous studies conducted on human subjects such as the one conducted by Stanglet al. (2011) have reported no correlation between lycopene plasma levels and FMD (Stangl et al., 2011).

### 10.4. Other Health Benefits

Lycopene is also capable of scavenging free radicals and has high health benefits for other disease conditions. It has been found that treating diabetic rats (streptozotocin-induced) with lycopene (1, 2 and 4 mg/kg) significantly improved oxidative–nitrosative stress, inflammation and cognitive deficits [100]. Increased memory with restored glutathione functioning was found in lycopene rats treated with 3-nitropropionic acid [101]. It has also been shown that cognitive impairment is elevated by low levels of lycopene in blood plasma [102].A recent study conducted by Crowe-White et al., 2019, has reported that three out of four studies conducted showa positive relationship between lycopene and maintained cognition; however, it also suggests that more thorough investigations are required to confirm the relationship between lycopene and cognitive longevity and dementia-related mortality (Crowe-White et al., 2019).

**Figure 2:**
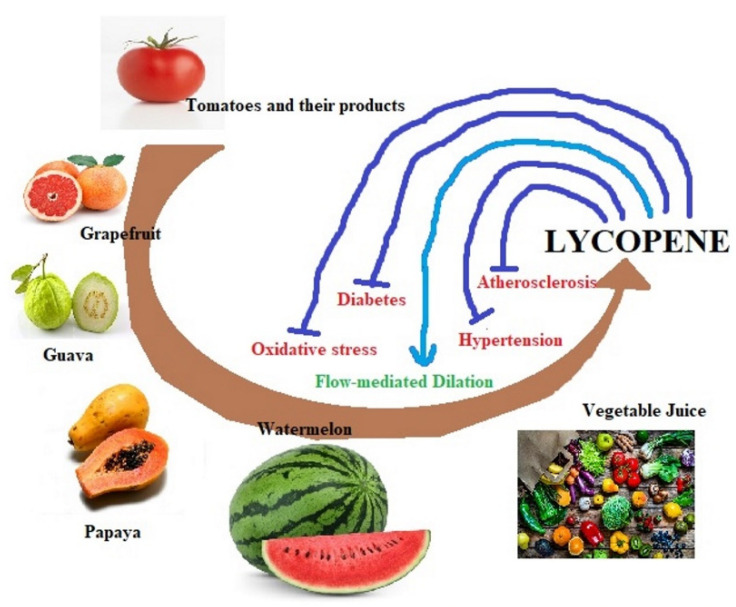
Overview of lycopene sources and its effect on cardiovascular disease factors.

**Figure 3:**
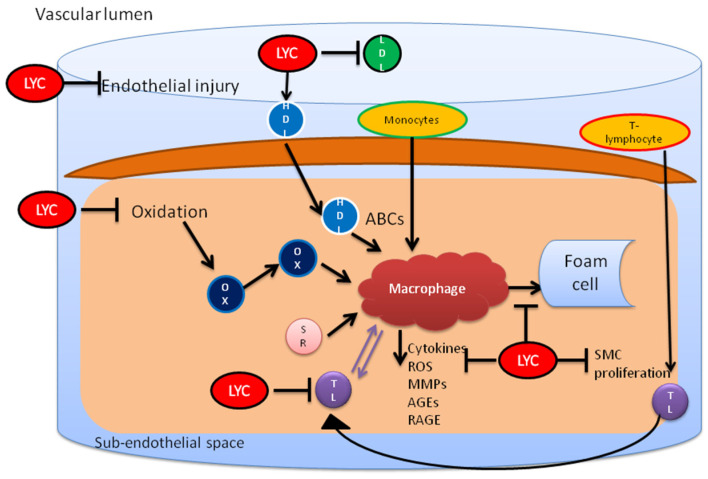
Lycopene inhibits endothelial injury and cholesterol by preventing oxidation of LDL. It also restores the functionality of HDL and prevents the proinflammatory activity initiated by T lymphocytes and macrophages. This leads to inhibition of foam cell formation from macrophages. LYC, lycopene; OX, oxidized HDL; MO, monocytes; ABCs, ATP-binding cassette transporters; MMP, metalloproteinases; AGE, advanced glycation end products; SMC, smooth muscle cells.

Thus, the protective effects of lycopene have been seen on CVD, hypertension, oxidative stress, cancers, diabetes, atherosclerosis, etc. However, the exact role of lycopene against these diseases is still unknown. Various effects of lycopene in relation to cardiac health are overviewed in Figure 2, while its influences on different signaling pathways are summarized in Figure 3.

## 11. Controversies and Associated Toxicity

A majority of studieshave proven the positive effects of lycopene on CVD, but many of them also failed to prove the consistent relationship of lycopene intake to a decrease in the risk of CVD, encompassing both prospective and retrospective studies and human interventional studies [75,103]. Among the many reasons for such negative relations, one reason is that the bio-availability and metabolism of lycopene are affected by genetic variability due to at least 28 SNP (single-nucleotide polymorphisms) in 16 genes [104]. In addition, the CVD markers used in the studies differ considerably, including biochemical parameters such as blood pressure and LDL levels and including clinical outcomes such as stroke, death and myocardial infarction, in which converging with a comparative result isdifficult. This variability is also found in the sources of lycopene such as fresh tomatoes, processed tomatoes, lycopene supplements, tomato-containing foods, etc. Discrepancies are also found in lycopene dosages, and consuming foods containing other sources may maximize or minimize lycopene distribution. Further, many studies used less than 100 volunteers, which minimizes the statistical power. It is therefore recommended that future studies should consider volunteers from a large population within the same geographical location in order to avoid genetic variability. In addition, it has been proposed that a wash out time of lycopene from previous ingestion should be given before starting the experimental procedure, as lycopene stays in the body for a considerable time [105].

The safety aspects of bioactive compounds are also kept on constant scrutiny by food scientists at all times to prevent any side effects. Lycopene, however, has been found to be safe (GRAS, Generally Recognized as Safe) either in its natural or synthetic forms when used as a food additive [106]. A direct developmental or maternal toxicity was found in rats and rabbits within a dosage of 2–3 g/kg/day administration of synthetic lycopene [107], and the safest level was found to be 75 mg/day for lycopene intake [108].

## 12. Conclusions

Thus, we conclude that lycopene plays a critical role in human health, particularly in preventing cardiovascular risks. Limited studies have been carried out in this area considering the consumption aspect of lycopene alone. The interactions of lycopene with other bioactive compounds are important for its bio-availability and distribution. However, more studies are required in this field to make nutritional strategies through food for preventing cardiovascular diseases.

## Figures and Tables

**Table 1:** Lycopene sources with processing method and lycopene content.

S.I	Food Source	Processing Method	Lycopene Content (µg/100 g)
1	Acai berry drink	Fortified beverage	899
2	Asparagus	Cooked	30
3	Cabbage (red/raw)		20
4	Grapefruit (red/pink)	Raw	1419
5	Grapefruit juice (red/pink)	Added Calcium	297
6	Guavas	Raw	5204
7	Guava nectar	Canned fortified with ascorbic acid	35
8	Guava sauce	Cooked	3909
9	Ketchup		12,062
10	Papayas	Raw	1828
11	Peppers (sweet, red)	Sauteed	484
12	Persimmons (Japanese)	Raw	159
13	Sapote (mamey)	Raw	199
14	Tomatoes	Sundried	45,902
15	Tomato products	Canned/puree without salt	21,754
16	Tomatoes	Crushed and canned	5106
17	Tomatoes (red/ripe)	Stewed, canned	4088
18	Tomatoes (red/ripe)	Cooked	3041
19	Vegetable juice (cocktail)	Canned	7119
20	Watermelon	Raw	4532

## Data Availability

Not applicable.
